# Molecular Views of Mineral Carbonation: Reaction of CO_2_ with the Wollastonite (100) Surface

**DOI:** 10.1021/acsnano.5c19629

**Published:** 2026-03-24

**Authors:** Andrea Conti, Luca Lezuo, Alexander Hoheneder, Elena Vaníčková, Domitilla Alessandra Aloi, Andreas Steiger-Thirsfeld, David Heuser, Rainer Abart, Florian Mittendorfer, Michael Schmid, Ulrike Diebold, Giada Franceschi

**Affiliations:** † Institute of Applied Physics, TU Wien, 1040 Vienna, Austria; ‡ Central European Institute of Technology, 27259 Brno University of Technology, 61200 Brno, Czech Republic; § University Service Center for Transmission Electron Microscopy, TU Wien, 1040 Vienna, Austria; ∥ Department of Lithospheric Research, Universität Wien, 1090 Vienna, Austria

**Keywords:** CO_2_, carbonate formation, AFM, DFT, wollastonite, surface chemistry, mineral surfaces

## Abstract

The carbonation of silicate minerals is a key process in the Earth’s carbon cycle and offers a promising avenue for long-term CO_2_ sequestration. However, the atomistic mechanisms by which CO_2_ is activated at silicate surfaces remain poorly understood, largely due to the intrinsic complexity and insulating nature of these materials. To close this gap, wollastonite (CaSiO_3_) is used as a model system. Noncontact atomic force microscopy (nc-AFM) with functionalized tips is combined with density functional theory (DFT) to investigate its lowest-energy (100) surface under ultrahigh vacuum (UHV). Upon cleaving the mineral in UHV, water vapor is released from the sample and spontaneously readsorbs into a previously unreported, exceptionally stable configuration. The resulting surface hydration layer promotes spontaneous CO_2_ chemisorption and the formation of surface carbonates with negligible kinetic barriers. Our results offer atomic-scale evidence of gas–phase carbonation on a silicate mineral, revealing a water-assisted pathway for CO_2_ capture that bypasses aqueous mineral dissolution.

## Graphical abstract

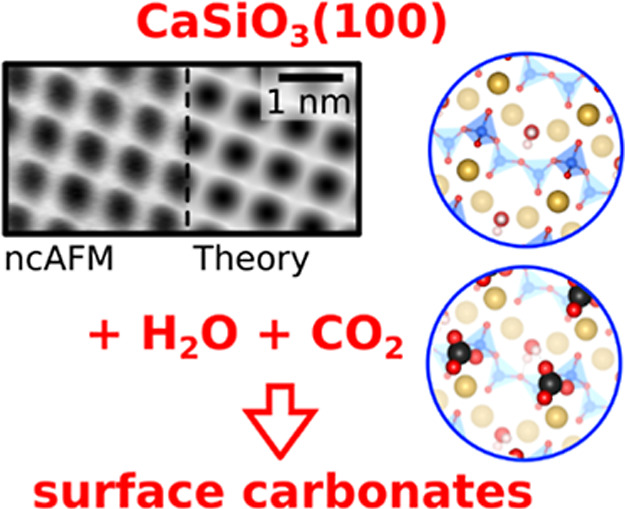

## Introduction

Gas–solid interfaces govern a wide range of processes across catalysis, geochemistry, and materials science. Whether in the catalytic conversion of molecules, the corrosion of materials, or the growth and weathering of minerals, surface reactivity is determined by the local atomic configuration. A mechanistic understanding of these processes requires tools capable of resolving how individual molecules interact with specific surface sites.

Progress in this area has been driven by surface science investigations under ultrahigh vacuum (UHV), which offer unparalleled control over surface composition, atomic structures, and gas exposure. Over the past decades, UHV studies have delivered critical insights into gas adsorption and reaction mechanisms on model surfaces through a combination of area-averaging spectroscopies, real-space scanning probe microscopy, and theoretical modeling. Among the many gases studied, carbon dioxide (CO_2_) is particularly significant. In natural environments, CO_2_ reacts with silicate minerals to form carbonates, leading to CO_2_ sequestration over geological time scales.^1^ Accelerating silicate carbonation through the injection of supercritical CO_2_ (scCO_2_) into deep geological formations is a promising strategy for climate mitigation.^2–5^ However, the atomistic pathways and the role of water underlying silicate carbonation in this context remain debated.^6^ Recent computational studies have proposed that the traditional dissolution–precipitation pathway (where CO_2_ forms HCO_3_^–^ and reacts with mineral-released ions) may be accompanied by direct surface reactions via CO_2_ chemisorption,^7,8^ leading to a much faster carbon capture as sluggish dissolution-based chemistry is avoided. Probing such a gas-phase mechanism is, in principle, more straightforward than solution-based pathways, yet the atomic-level details remain difficult to unravel. As a chemically inert, linear molecule, CO_2_ must first be activated, typically through bending, charge transfer from the substrate, and new bonds forming with surface atoms.^9^ This activation depends sensitively on the local surface environment: coordination geometry,^7^ surface basicity,^10^ the presence of defects, and coadsorbates like water all significantly influence reactivity.^6,7^ Even on well-defined single crystals, these factors are often intertwined, leading to multiple adsorption geometries and reaction pathways.

Disentangling these effects to understand how CO_2_ adsorbs and becomes activated at surfaces has long been a central goal of UHV surface science.^9,11,12^ Among the various approaches, vibrational spectroscopy has played a key role in identifying binding modes, particularly on metals and a few binary oxides used as model substrates. These studies have revealed a diverse range of CO_2_ adsorption configurations. On binary oxide single crystals—the closest analogs to natural minerals that have been systematically probed—CO_2_ typically remains linear and physisorbed unless activated at defects or undercoordinated sites.^13–17^ In rarer cases, more reactive species, such as bent CO_2_^δ−^, carboxylates, and surface carbonates, have been identified.^18–22^ While vibrational spectroscopy has provided fingerprints of carbonate and bicarbonate species,^11^ the spectral interpretation under realistic conditions is often complicated by overlapping features in the C–O stretching region^23,24^ and by the interplay with water, which can either block reactive sites or promote CO_2_ activation via hydroxyl-assisted pathways.^13,25,26^ These coupled effects underscore the need for complementary, local probes that can resolve individual adsorption geometries and reactive sites. However, such atomic-scale investigations have remained rare,^14,17^ especially for silicate minerals that are electrically insulating.^27–30^ Computational studies of their carbonation have advanced much further than experimental investigations.^7,31–38^

To address these issues, UHV-based surface science is turning to increasingly sophisticated techniques. Noncontact atomic force microscopy (nc-AFM) has emerged as a powerful tool for resolving surface structure and chemistry of various samples, including insulators, at the atomic level. The combination of qPlus sensors^39^ and functionalized tips, such as CO-terminated tips for molecular resolution^40^ or O-terminated tips for oxides,^41^ now enables real-space imaging with intramolecular resolution and chemical sensitivity.^42–48^ These advances open new possibilities for probing the surface chemistry of geochemically relevant materials otherwise inaccessible to traditional surface science methods.

Among candidate minerals for nc-AFM investigations, wollastonite (CaSiO_3_), a naturally occurring calcium silicate, stands out for its high reactivity toward CO_2_.^2,49–55^ Its carbonation potential has been widely studied in solution and under high-pressure CO_2_ atmospheres, where it readily undergoes reactions to form stable calcium carbonate phases. These properties are being harnessed in varied applied contexts such as soil amendment—where Ca-rich silicates improve nutrient availability while promoting CO_2_ sequestration^56,57^ —and the development of carbon-neutral cements as alternatives to traditional Portland-based formulations.^55,58^ Despite the growing interest, the fundamental origin of wollastonite’s high reactivity remains unclear. It is still debated whether its superior CO_2_ uptake arises from particularly fast dissolution kinetics, favorable precipitation dynamics,^3^ or the possibility of a direct, dry-gas carbonation pathway.^8^ Resolving this question requires an atomistic understanding of how CO_2_ interacts with wollastonite surfaces. Theoretical studies have proposed that low-index wollastonite surfaces can activate CO_2_ via tilted adsorption geometries that facilitate charge transfer and bond formation.^8,32^ However, predicted adsorption motifs and surface terminations vary between models,^32,59^ and direct experimental atomically resolved validation on a well-defined single-crystalline surface has been lacking.

Here, we combine nc-AFM imaging under UHV conditions with density functional theory (DFT) calculations and corresponding AFM simulations based on the Probe-Particle Model^60^ to investigate the (100) termination of wollastonite and its reactivity toward CO_2_. Upon cleaving, water vapor released from within the sample rapidly hydrates the surface, with one water molecule per unit cell present in a highly stable, previously unreported adsorption configuration. These water molecules enable spontaneous CO_2_ chemisorption and the formation of a surface carbonate with a binding energy of −0.84 eV. Without coadsorbed water, by contrast, CO_2_ remains weakly adsorbed and unreactive.

## Results

### Cleaved, Hydrated Wollastonite Surface

A photograph of the wollastonite specimen is shown in Figure 1B. It consists of fibrous grains elongated along their [010] direction of up to 1 cm in length and 1 mm in width. More details about its macroscopic characterization (X-ray diffraction (XRD), electron probe microanalysis, polarization microscopy, and electron backscattered diffraction (EBSD)) are provided in the Methods Section and Section S2. When cleaving a grain, well-defined terraces become visible to the naked eye and in optical microscopy (Figure 1C). These terraces are elongated along the [010] direction and appear optically flat within the cleavage (100) plane, as identified by EBSD (Figures S3 and S5). This macroscopic morphology is mirrored at the mesoscale, as revealed by ambient AFM (Figure 1D): The surface features atomically flat terraces aligned predominantly along [010], separated by monatomic steps (≈750 pm) or multiples thereof. Terrace widths range from a few to several hundred nanometers (see Figure S4 for additional AFM data). Atomically resolved nc-AFM imaging in UHV performed within a single terrace reveals a flat and periodic surface structure (Figure 1E), characterized by a rectangular unit cell of ≈7.3 Å × 7.0 Å, consistent with the lowest-energy (100) cleavage, as further supported by the analysis presented in Section S1. The surface is sparsely decorated with point defects of currently unknown nature, occupying ≈2% of the lattice sites. The following analysis focuses on defect-free regions. The UHV-cleaved sample showed no contaminants within the detection limit of X-ray photoelectron spectroscopy (XPS, Figure S6) except for carbon, whose presence might be due to uncleaved portions of the needles, the glue used for mounting (see Methods Section), or small fractions of calcite (CaCO_3_).

**Figure 1:**
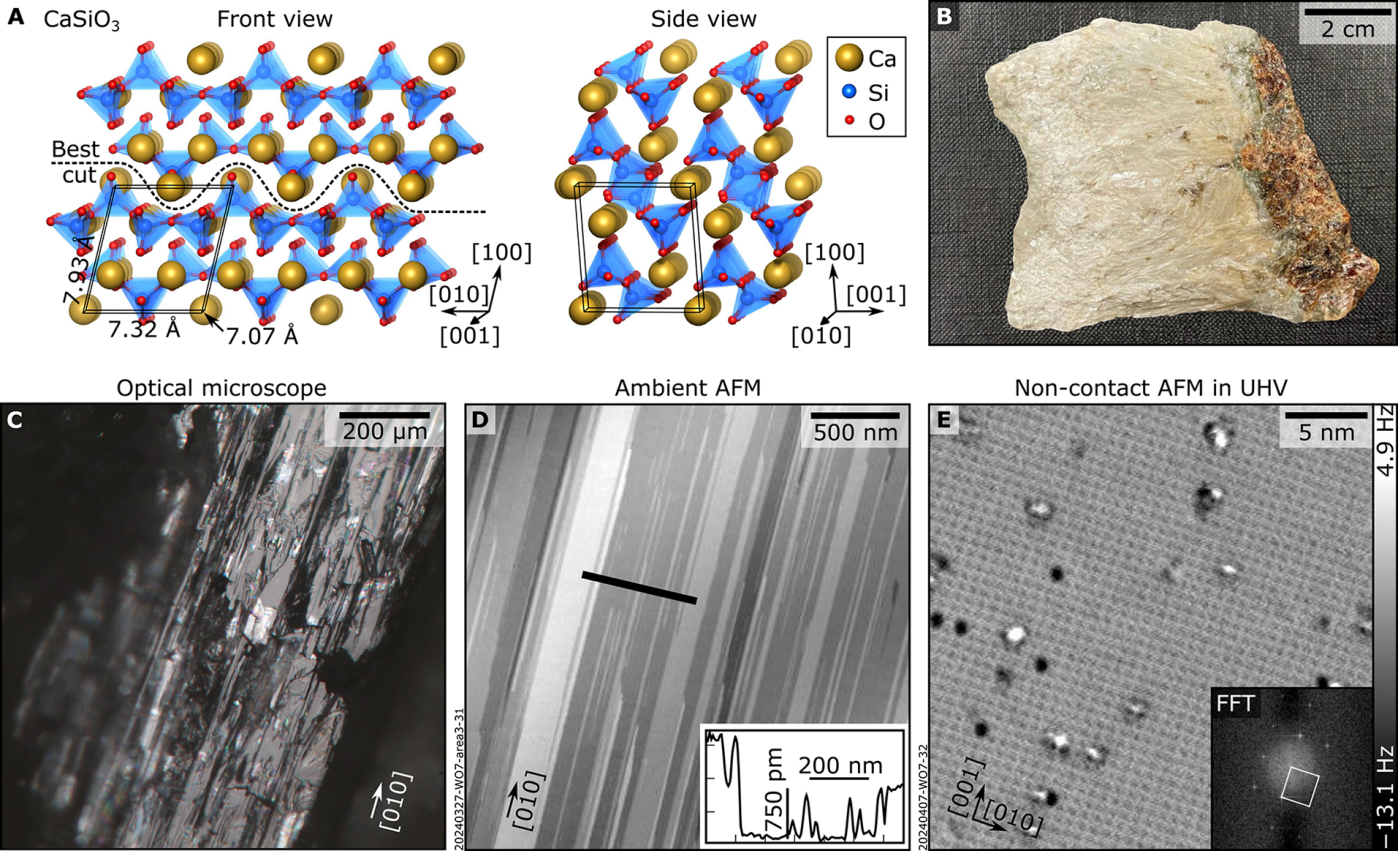
(100) surface of wollastonite. (A) DFT-optimized structure of the bulk (front and side views). The front view shows the chains of corner-sharing SiO_4_ tetrahedra running along the [010] direction and indicates the best cleavage plane. (B) Hand specimen of wollastonite aggregate from a skarn deposit in Turkey used in this work. Macroscopically, the wollastonite appears as white and transparent needle-, lath-, and rod-shaped grains. On the right side, a darker layer of red garnet and green clinopyroxene grains is visible. The wollastonite grains are elongated subperpendicular to this layer. (C) Reflected-light optical microscopy image of a (100)-oriented, cleaved wollastonite grain. (D) 2.5 × 2.5 μm^2^ ambient AFM image on such a grain, with the height profile along the black line in the inset. (E) 27 × 27 nm^2^ nc-AFM image of wollastonite (100) cleaved in UHV (tip amplitude *A* = 200 pm, sample voltage *V*_s_ = −10 V; acquired at 5.7 K with a Cu-terminated tip). Point defects are visible on an otherwise periodic surface. Inset: fast Fourier transform (FFT).

The observed surface morphology is rooted in the bulk crystal structure of wollastonite (Figure 1A), which is triclinic (space group P1̅). Isolated undulating chains of corner-sharing SiO_4_ tetrahedra run along [010]. The tetrahedra are linked via apical oxygen atoms, while Ca^2+^ ions reside in the interchain regions, coordinated with oxygen atoms from the adjacent chains. This structural anisotropy helps explain the natural (100) cleavage that yields terraces elongated in [010]. From a theoretical standpoint, two primary cleavage possibilities have been proposed in the literature. Luan et al. proposed a cleavage that involves breaking SiO_4_ tetrahedra.^32^ In contrast, Kundu et al. predicted cleavage through a plane made of Ca^2+^ ions that avoids breaking Si–O bonds.^59^ DFT calculations conducted in the current work support the latter, indicating a substantially lower surface energy for the Ca^2+^ plane (58 meV/Å^2^) compared to the one proposed by Luan and Dholabhai,^32^ i.e., 110 meV/Å^2^, even after a significant reconstruction to compensate for the undercoordinated Si atoms. Thus, the pristine cleaved surface is expected to expose a bulk-truncated termination characterized by Ca^2+^ ions and intact SiO_4_ units arranged in a rectangular lattice—consistent with the periodicity observed by nc-AFM (Figure 1E). Note that such a pristine, “dry” surface, however, was not probed experimentally in this work. Akin to other silicate minerals,^28,61^ water that had been trapped within the mineral during its growth is released upon cleavage, as confirmed by mass spectrometry (Figure S7). As discussed in the following sections, this water vapor readily interacts with the cleaved surface, leading to spontaneous surface hydration.

To probe the role of cleavage-released water, DFT calculations examined the interaction of water with wollastonite (100). Previous computational studies have suggested that molecular adsorption occurs with the oxygen of the H_2_O binding to a surface Ca^2+^ ion and a hydrogen bond with the apical oxygen of a nearby SiO_4_ tetrahedron.^59^ In this “protruding” configuration, H_2_O resides above the nearly bulk-truncated surface with an adsorption energy of −0.97 eV per molecule, as determined from our first-principles calculations. The simulations performed in the present work, however, find a different and more favorable adsorption geometry. The first H_2_O molecule adsorbs without a barrier in the grooves formed by the downward-pointing SiO_4_ tetrahedra created upon cleaving—a configuration henceforth referred to as “nested” H_2_O (see Figure 2A for the DFT-optimized structure). In this arrangement, H_2_O is coordinated to two subsurface Ca^2+^ ions via Ca–O bonds and donates a hydrogen bond to a bridging oxygen atom located between two SiO_4_ tetrahedra. The calculated adsorption energy, −1.3 eV, is unusually strong for molecular H_2_O—comparable only to structures including dissociated H_2_O on other oxides known to behave as Brønsted bases, such as In_2_O_3_(111)^62^ and ZnO(101̅0)^63^—and indicates that this configuration is thermodynamically favored and stable at temperatures up to 100 °C in UHV.^64^ Thus, one expects that water, released while cleaving at room temperature in UHV, will readily adsorb on wollastonite (100) into the nested configuration. Based on mass spectrometry data (Figure S7), the amount of water liberated during cleavage should be sufficient to saturate all nested adsorption sites. Additional water, if present in excess, is unlikely to adsorb at room temperature: the binding energy for the next available configuration (the “protruding” geometry predicted by Kundu et al.) is substantially weaker,^59^ and therefore unfavorable in vacuum at ambient temperatures. Note that hydroxylation is also unfavorable: dissociated water reverts to molecular water even with standard DFT relaxations. This is different from the predicted behavior of the more reactive, high-surface-energy (001) facet of wollastonite, where water is expected to dissociate during chemisorption.^8,65^ Annealing the samples to induce the desorption of the nested water was not possible since the glue needed for mounting the samples (see Methods) strongly degasses above 150 °C and induces surface contaminations even at lower temperatures.

**Figure 2:**
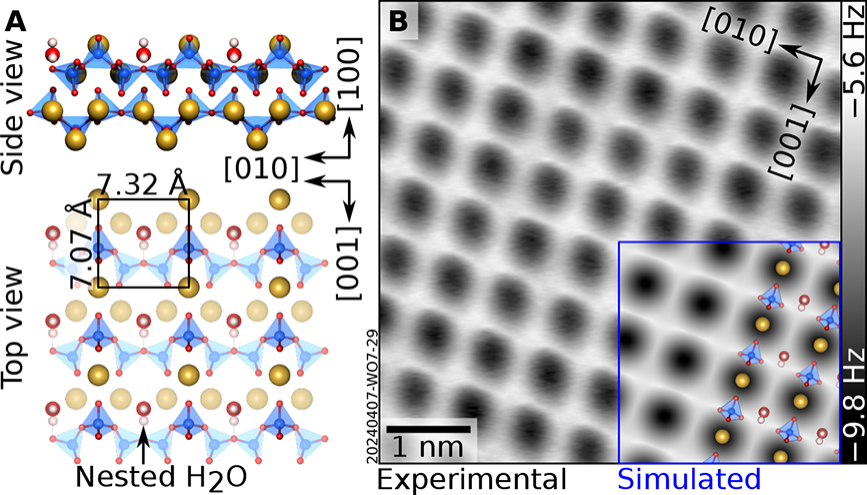
Cleaved, hydrated wollastonite (100). (A) DFT-optimized surface structure of wollastonite (100) with one H_2_O per unit cell adsorbed in the “nested” configuration (see text). (B) 5.5 × 5.5 nm^2^ nc-AFM image of cleaved wollastonite (100) that has reacted with H_2_O at room temperature (*A* = 200 pm, *V*_s_ = −10 V; acquired at 4.7K with a Cu-terminated tip). Dark gray indicates attractive tip–surface interaction, while tip–sample interaction is weak in the light-gray regions. The overlay shows an AFM simulation derived from the DFT model on the left-hand side (tip–sample distance of 4.8 Å).

Figure 2B shows a close-up view of the cleaved surface acquired by nc-AFM with a Cu-terminated tip. The image reveals a rectangular array of dark dots, whose contrast becomes increasingly pronounced—larger and darker—at reduced tip–sample distances (Figure S9). The contrast is well reproduced by AFM simulations (overlay with blue frame in Figure 2B) based on the DFT-optimized structure of Figure 2A. The dark dots are attributed to surface Ca atoms, while the thin, bright lines along [010] run between the two uppermost O atoms of the surface SiO_4_ tetrahedra. When using oxygen-terminated tips, the O atoms appear bright (repulsive)—a contrast also well captured by AFM simulations (Figure S9). FFT analysis (see Figure 1E) confirms that the measured surface periodicity is consistent with the unit cell predicted by DFT for the bulk-truncated (100) surface. Importantly, because the adsorbed water molecule in the nested position lies below the topmost Ca and O, it is invisible in constant-height nc-AFM imaging. Indeed, simulated AFM images for both pristine and hydrated surfaces are indistinguishable (Figure S12). Nevertheless, nested water has a measurable effect on CO_2_ adsorption, as shown in the following.

### Carbonate Formation

Figure 3 summarizes the results of CO_2_ adsorption on the hydrated wollastonite (100) surface. Nc-AFM images reveal isolated features that increase in number with longer exposure to CO_2_ (see Figure S10 for coverage-dependent data). The observed contrast varies markedly with tip termination: CuOx tips (Figure 3B,C) reveal the adsorbates as bright (repulsive) features above the darker Ca layer, whereas Cu- and CO-terminated tips show them dark (attractive; Figure S11). Some features can be manipulated by the tip below a critical tip–sample distance (arrows in Figure 3B).

**Figure 3:**
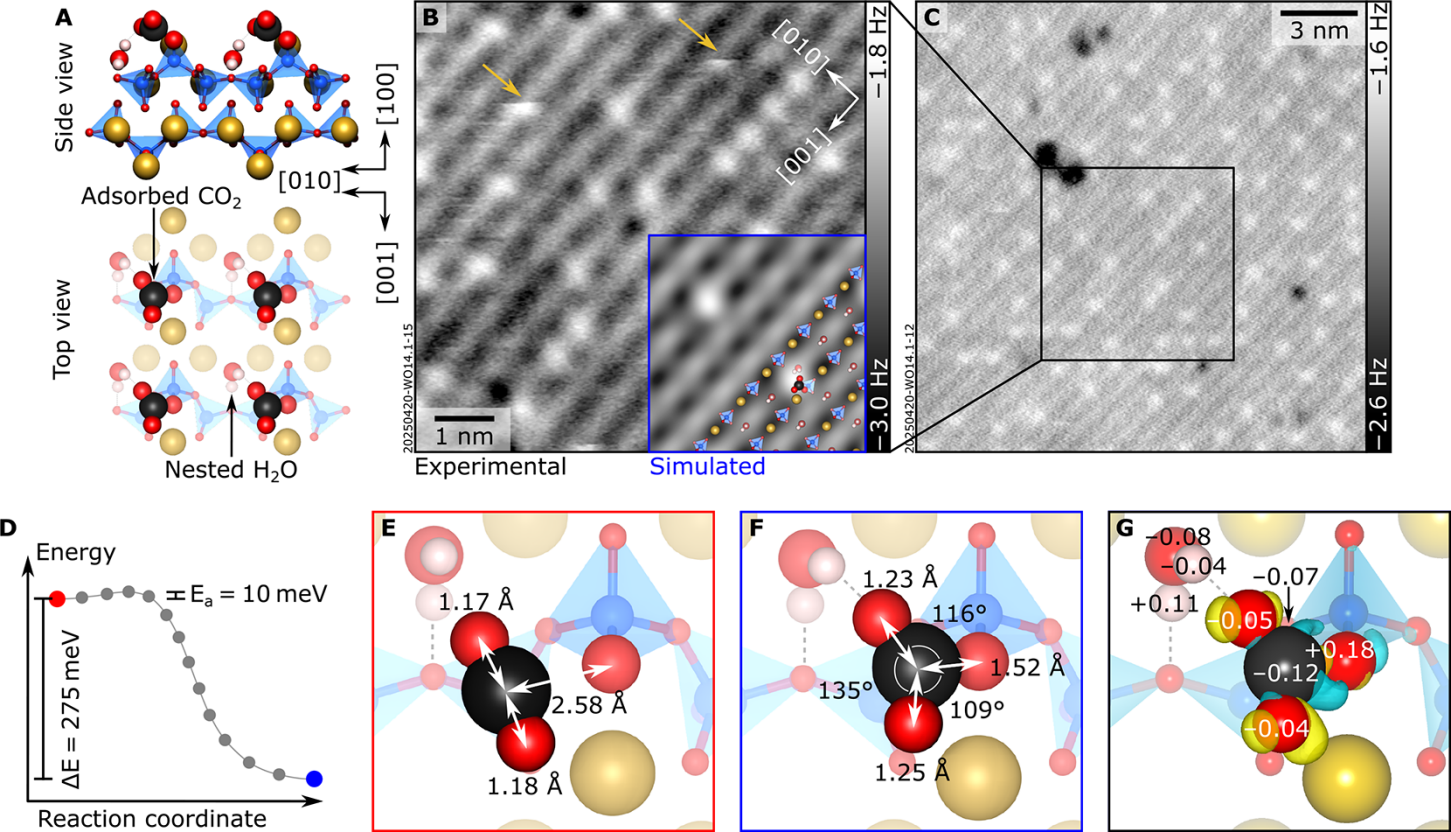
Carbonate formation on hydrated wollastonite (100). (A) DFT-optimized structure of hydrated wollastonite (100) with adsorbed CO_2_. (B,C) 9 × 9 nm^2^ and 19 × 19 nm^2^ nc-AFM images (*A* = 700 pm, *V*_s_ = −4 V; acquired at 78 K with a CuOx tip). The overlay in panel B shows an AFM simulation derived from the DFT model on the left-hand side (tip–sample distance of 5.8 Å, partial CO_2_ coverage). Yellow arrows indicate tip-induced manipulation events. (D) Minimum-energy profile for CO_2_ adsorption plotted as total energy versus the reaction coordinate. The red circle denotes the initial, physisorbed state (panel E), the blue circle the final, chemisorbed state (panel F), and the gray dots intermediate configurations along the nudged-elastic-band pathway. (E,F) Top view of the DFT-optimized models of the initial physisorbed and final chemisorbed states, respectively. Bond lengths (double arrows) and angles are indicated; dotted lines indicate H bonds. (G) Bader analysis and plot of the charge density difference between the adsorbed and isolated (bent) CO_2_ molecule on hydrated (100) wollastonite. Bader charge differences, before and after CO_2_ chemisorption, are indicated in elementary charges and only if the absolute value is larger than 0.03 e (see Figure S13 for the complete Bader charge analysis). The overlaid surfaces (isovalue 0.01 e/Å^3^) denote electron density accumulation (yellow, charge gain) and depletion (cyan, charge loss), respectively.

DFT calculations predict that CO_2_ chemisorbs at the hydrated surface with a negligible activation barrier (10 meV, see Figure 3D), meaning that the reaction can proceed even at cryogenic temperatures in UHV. The computed adsorption energy of −0.84 eV suggests that CO_2_ should be stable at ≈200K in a low-pressure environment but should desorb upon heating to room temperature in UHV.^64^ Chemisorption involves multiple interactions: an H–bond with the protruding H of the nested H_2_O (bond length: 1.92 Å), a Ca–O bond to a surface Ca^2+^ site (bond length: 2.36 Å), and a covalent bond from the carbon to the apical oxygen of a surface SiO_4_ tetrahedron (bond length: 1.52 Å). This bonding geometry induces a pronounced bending of the originally linear CO_2_ molecule (Figure 3F).

There is no sharp criterion to distinguish whether the adsorbed species should be considered an activated CO_2_ or a surface-bound carbonate created by interaction with the substrate. The species shown in Figure 3F can be named a carbonate based on its geometry: The adsorbed CO_2_ molecule adopts a bent configuration with O–C–O angles of 135°, 109°, and 116°. This is far from the 180° of linear CO_2_ and much closer to the bond angles of a gas-phase CO_3_^2–^ ion (120°). The calculated C–O bond lengths (1.23 Å, 1.25 Å, and 1.52 Å) tend toward those of a gas-phase carbonate species (CO_3_^2–^), which has C–O bond lengths of ≈1.26 Å with the DFT functional used in this work rather than those of neutral CO_2_ (≈1.16 Å). Furthermore, the density of states (DOS) in Figure S14 reveals new C–O hybridized states around −20 eV and between −10 and 0 eV, absent in the physisorbed case. This is consistent with covalent bond formation between the C atom of the CO_2_ molecule and the surface O of the SiO_4_ tetrahedron (Figure S14B). Bader charge analysis (Figures 3G and S13) reveals a net electron transfer from the substrate to the CO_2_. The charge loss between the C atom and the surface O shows characteristic “wing-like” features reflecting the breaking of the original C=O π bonds.

Critically, the signatures of carbonate formation only emerge in the presence of nested water. When a calculation of the configuration of Figure 3A is run after removing the nested H_2_O and relaxing the rest of the structure, the covalent C–O bond is lost, and the molecule goes back to a physisorbed state. AFM simulations for CO_2_ on the hydrated model reproduce the observed experimental contrast for three tip terminations, CO and Cu (Figure S11), and CuOx (Figure 3B), further validating the proposed structure. In contrast, in the absence of nested water, CO_2_ adsorbs in the “nested” site with a reduced binding energy (−0.75 eV/CO_2_), stabilized by Ca–O interaction with subsurface Ca atoms (Figure S12D). In this configuration, the molecule lies flat and would be undetectable by nc-AFM (left inset in Figure S12G). At higher coverage on the water-free surface (2 CO_2_ per unit cell), an additional CO_2_ can bind between two surface Ca atoms in a planar geometry with a weaker binding energy (−0.51 eV/CO_2_, Figure S12F), producing a starkly different AFM signature (right inset in Figure S12G). Thus, both experiments and theory converge to show that CO_2_ activation on wollastonite (100) is mediated by surface hydration: The presence of the nested water molecule is indispensable for stabilizing the carbonate. Without it, CO_2_ remains weakly bound and unreactive.

## Discussion

Over the past decades, numerous studies have sought to rationalize mineral carbonation across diverse environmental and engineered settings.^3,6^ Traditionally, mineral carbonation has been described by the so-called dissolution–precipitation pathway,^66^ in which mineral surfaces interact strongly with the ubiquitous water: following dissolution, minerals release ions that react with dissolved bicarbonate species to form stable carbonates. However, this framework does not fully account for observations in emerging carbon capture and storage strategies characterized by a CO_2_-rich atmosphere, such as scCO_2_ injections. The mineralization rates measured in field scCO_2_ experiments (conversion rates of 60% within two years^67^) have been significantly underestimated by numerical models based solely on dissolution–precipitation (conversion rates between 17% and 60% after 150 years^68^). A possible explanation is offered by a recent computational study on basaltic rocks.^7^ Under scCO_2_ conditions, the dominant reactive species at high pressure is not aqueous bicarbonate, but CO_2_ itself. In this scenario, mineral surfaces may still be hydroxylated or hydrated, but due to the lack of a liquid aqueous solution, carbonation by dissolution–precipitation cannot occur. Instead, CO_2_ may undergo direct chemisorption at the water-reacted surface, giving rise to carbonate, bicarbonate, or hydrogen pyrocarbonate species depending on the silicate mineral, its termination, and its previous interaction with water. Such reacted surface species may, in turn, promote subsequent mineral dissolution. Since dissolution is the rate-limiting step of mineral carbonation,^4^ the existence of this alternative chemisorption-assisted pathway could help explain the high mineralization rates observed in the field.^7^ Other computational works have proposed similar chemisorption pathways for various water-modified mineral surfaces.^8,35,36,38^

The present work demonstrates the existence of such a pathway on a well-defined surface of single-crystalline wollastonite, a calcium silicate mineral with high relevance for CO_2_ sequestration, complementing studies on related systems investigated on powder samples or on prepared (polished and sputtered) mineral surfaces.^8,69^ The adsorption of water in the “nested” configuration observed in UHV is expected to occur at conditions relevant to scCO_2_ injections (reservoirs below 50 °C)^70,71^ due to the barrierless reaction observed in DFT and the strong adsorption energy (−1.3 eV), enabling the proposed carbonation reaction. Interestingly, the density of surface carbonates found on wollastonite (100) (1 molecule per (1 × 1) surface unit cell, corresponding to 1.93 molecules nm^–2^) exceeds the values predicted for minerals in basaltic rocks (0.25–1 molecules nm^–2^), where chemisorption has been estimated to contribute up to 20% of the total storage potential per year.^7^ This suggests that wollastonite may offer particularly favorable conditions for CO_2_ uptake under high-pressure scenarios such as scCO_2_ injection. Importantly, our measurements were performed on well-ordered, defect-poor surface regions imaged by nc-AFM. Since surface defects generally increase reactivity by providing undercoordinated sites, the true carbonation capacity of natural, defect-rich wollastonite surfaces is likely higher.

Notably, both this study and previous literature show that the pathways leading to carbonation are highly system-specific. In basaltic minerals explored computationally, the density and type of surface oxygen atoms as well as the degree of hydroxylation lead to starkly different outcomes—ranging from carbonate to bicarbonate or pyrocarbonate formation—and thus to strongly varying carbonation rates across different minerals.^7^ Similarly, wollastonite demonstrates distinct behaviors on its (100) and (001) terminations: on the (100) surface investigated here, water remains molecular and promotes carbonate formation, whereas on (001) surfaces, water is predicted to dissociate and hydroxylate the surface,^65^ thereby inhibiting carbonate formation rather than promoting it.^8^ Such contrasting behaviors underscore the central role of water in governing gas–solid carbonation and emphasize that reactivity depends sensitively on the surface atomic structure. Our results therefore complement the growing body of computational studies on silicates under scCO_2_ injection conditions,^37^ by providing direct experimental evidence of a water-mediated carbonation pathway. Taken together, these efforts highlight the need for systematic, surface-specific investigations across the minerals of the silicate family to build predictive models of carbonation kinetics relevant to carbon capture and storage.

## Conclusions

This study establishes wollastonite, a naturally occurring calcium silicate, as an experimentally accessible model system for probing the atomistic mechanisms of mineral carbonation. By combining noncontact atomic force microscopy with density functional theory, a structural model for the (100) surface termination is established, invoking a previously unreported hydration layer to rationalize the experimental observations. This hydration layer forms spontaneously upon cleavage under UHV conditions as water is released from the bulk and is expected to remain stable under ambient conditions. It plays a decisive role in promoting CO_2_ activation, enabling adsorption in a tilted, carbonate-like geometry with a negligible kinetic barrier through binding to a surface oxygen atom.

The findings provide experimental, atomic-scale evidence for a water-mediated gas–solid carbonation pathway on a silicate surface relevant in the context of CO_2_ sequestration. They complement and validate recent theoretical predictions that highlight the importance of direct chemisorption routes alongside the classical dissolution–precipitation mechanism, particularly under CO_2_-rich conditions such as in the context of scCO_2_ injections.

More broadly, the results demonstrate that atomic-scale studies of insulating, geochemically relevant minerals are now within experimental reach. By identifying the structural and chemical factors that enable carbonate formation, this work delivers the mechanistic insight needed to model mineralization kinetics from first-principles and illustrates how modern surface science approaches can address longstanding questions in geochemistry and CO_2_ capture at the molecular level.

## Methods

### Wollastonite Sample, Ex-Situ Characterization and Methods

The sample was extracted from a rock specimen of several centimeters in size from a skarn occurrence in Turkey (Figure 1B). The specimen is primarily composed of white or colorless, up to 1 cm-long and up to about 1 mm-wide, prismatic and fibrous wollastonite grains. On one side (right in Figure 1B), the sample comprises an about 1 cm thick, relatively dark layer with millimeter-sized isometric grains of green diopside-rich clinopyroxene and red andradite-rich garnet. The wollastonite grains show preferred shape orientation subperpendicular to the diopside-garnet layer.

Thin sections of 30 μm thickness were investigated by optical polarization microscopy using a Leica DM4500P polarization microscope. Optical microscopy images are shown in Figure S1, where lath-shaped wollastonite and fibrous wollastonite can be discerned; the former prevails in the central portion of the rock specimen represented by the wollastonite-rich zone, while the latter dominates at the contact of the wollastonite-rich zone to the garnet-diopside zone as well as at the opposite side of the sample. Within the wollastonite-rich zone, some grains of diopsidic clinopyroxene are present (<10 vol %). Within the diopside-garnet zone, calcite is present as xenomorphic grains. Minor calcite is also present in the zone of fibrous wollastonite on the opposite side of the sample.

The chemical composition of the wollastonite was determined by electron probe micro analysis using a CAMECA SX Five FEG instrument in the EPMA laboratory of the Core Facility Electron Beam Microanalysis, Faculty of Earth Sciences, Geography and Astronomy at the University of Vienna (AT), using 15 kV acceleration voltage and 20 nA beam current. Calibration for quantitative analyses was done on well-characterized mineral standards. The analysis revealed that the wollastonite is nearly pure CaSiO_3_ with >99 atom % wollastonite end-member fraction. The most prominent impurities are 0.17 wt % FeO, 0.06 wt % MgO, and 0.04 wt % MnO. Accordingly, the sum formula can be written as (Ca_0.996_Mg_0.002_Mn_0.001_Fe^2+^_0.001_)­[Si_0.999_ Fe^3+^_0.002_ O_3_].

Crystal structure characterization was done by powder X-ray diffraction (pXRD) on a Bruker D8 Eco diffractometer at the Department of Mineralogy and Crystallography, University of Vienna using Cu Kα radiation, for the range 2° < 2θ < 92°, with a Lynx-XET detector set to 4° 2θ angular opening, 0.01° 2θ step size and a counting time of 0.5 s per step resulting in an overall measuring time of 2 h. Peak and phase identification were performed using the DIFFRAC.EVA software (version 4.2) and the ICDD Powder Diffraction File PDF-2. For the extraction of unit cell parameters, Rietveld refinements were performed using the TOPAS-6 software. The measurements revealed that the specimen is triclinic type 1A wollastonite with lattice parameters *a* = 7.9258 Å, *b* = 7.3202 Å, *c* = 7.0653 Å, α = 90.055°, β = 95.217°, γ = 103.426° (see Section S2) and with needles preferentially oriented along (010).

Small needle aggregates were extracted from the rock sample and cleaved both inside the UHV chamber and in air. Their orientation was assigned a-posteriori by EBSD and nc-AFM to (100) (see details below and Figure S3 for EBSD; see Section S1 for geometric considerations based on nc-AFM). For the mounting, the grains were glued onto Omicron-style stainless-steel plates using UHV-compatible epoxy glue (EPO-TEK T7110-38), and a metal stud was glued to the top of the sample. Applying a tangential force to the stud enables the cleaving of the portion of the sample initially covered by the stud (see Figure S5). The mesoscale morphology of the grains cleaved in air was investigated with ambient AFM, specifically with an Agilent 5500 ambient AFM in intermittent contact mode with Si tips on Si cantilevers.

To determine the orientation of the cleaved wollastonite surfaces, one of the samples characterized by nc-AFM was examined by scanning electron microscopy (SEM) using a FEI Quanta 250 FEG operated at 20 kV with a probe current of ≈7.2 nA under low vacuum conditions. The exact terrace cleaved in UHV and imaged by nc-AFM was identified and targeted for EBSD measurements (Figure S3). EBSD data were acquired with an EDAX Hikari XP2 camera. The Kikuchi patterns underwent postprocessing with dynamic background subtraction, contrast enhancement, and neighbor pattern averaging and reindexing (NPAR). The orientation data were evaluated using spherical indexing with the corresponding material file.

### UHV Setup

The experiments were carried out in a UHV setup consisting of two interconnected chambers: A preparation chamber (base pressure below 1 × 10^–10^ mbar) for sample cleaving and X-ray photoelectron spectroscopy (XPS), and an adjacent chamber for nc-AFM (1 × 10^–11^ mbar).

The samples were cleaved at room temperature by grabbing the stud with a wobble stick while rotating the manipulator by a few degrees. During the cleaving, the residual-gas pressure always spiked by a few orders of magnitude, similar to the behavior when cleaving feldspars;^28^ the composition of the released gas was investigated by mass spectrometry (Figure S7). After cleaving, the samples were irradiated for 1 min with X-rays from the XPS setup to remediate the charge developed during UHV cleaving.^28^ An optical microscope attached to the UHV chamber helped identify the portions of the cleaved sample flat enough to approach the nc-AFM tip and acquire images (Figure S5).

CO_2_ (Linde 4.5 HiQ MINICAN) was dosed from a leak valve while keeping the sample on the preparation-chamber manipulator at 100 K. The number of molecules deposited is always expressed with respect to the surface unit cell on (100), i.e., (7.07 Å × 7.32 Å ≈52 Å^2^). Warming up the sample to room temperature caused the desorption of all CO_2_ molecules from the surface, as evidenced by nc-AFM.

XPS was performed with a nonmonochromatic dual-anode Mg/Al X-ray source (SPECS XR 50) and a hemispherical analyzer (SPECS Phoibos 100). Spectra were acquired with Al Kα radiation in normal emission. The binding energy axes were adjusted to account for shifts due to charging by taking the C 1*s* peak as reference (285 eV). The AFM measurements were performed at 4.7 and 78 K using a commercial Omicron qPlus LT head and a differential cryogenic amplifier,^72^ in constant-height mode. The qPlus AFM sensors (*k* = 2000–3500 N/m, *f*_0_ ≈27 or 32 kHz, *Q* ≈ 14,000 or 12,000) had a separate contact for the tunneling current. Tips obtained from an etched W wire were glued to the qPlus sensor and then prepared for measurement in UHV. Cu-, CO-, and CuOx-terminated tips were prepared on the oxygen-induced reconstruction of Cu(110) by repeated indentation and voltage pulses^41^ to exhibit a frequency shift smaller than −1.5 Hz. The tip terminations were identified in STM mode using characteristic fingerprints (Figure S8) and subsequently used for the unambiguous assignment of cationic and anionic surface species. This assignment is further supported by imaging at different tip–sample distances and by comparison with AFM simulations (see below). Local contact potential difference measurements by the Kelvin parabola method^73^ were performed to assess residual fields. Residual surface charges were compensated by applying a bias voltage *V*_s_ to the back of the sample plate while keeping the tip potential close to ground.

AFM images were processed with ImageJ:^74^ Raw images were corrected for distortions due to piezo creep^75^ and filtered in the Fourier domain to remove mechanical and electrical noise. AFM calibration was carried out using well-established reference surfaces. The vertical (z) calibration was performed by comparing measured step heights with their known values, while the lateral (x and y) calibration was obtained from fast Fourier transform (FFT) analysis of the AFM images.

### Computational Methods

DFT calculations were performed using the projector-augmented wave (PAW) method,^76,77^ as implemented in the Vienna Ab-initio Simulation Package (VASP, version 6.5.1),^78^ using the metaGGA r^2^SCAN + rVV10 ^79–81^ exchange–correlation functional. This functional provides a good description of the bulk properties, with lattice parameters and angles deviating by less than 0.5% from the experimental values measured by XRD. The bulk structure was optimized with a cutoff energy of 800 eV, using a 3 × 3 × 3 k-point mesh to integrate the Brillouin zone and a Gaussian smearing with a width of 0.1 eV. A lower cutoff energy of 500 eV was employed for the calculations of the (100) surface with a 1 × 3 × 3 k-point mesh. The slabs were symmetric, with a thickness of 3 bulk unit cells (i.e., 18 formula units of CaSiO_3_, 90 atoms in total), and separated by 15 Å vacuum spacing. All atoms were free to relax. The geometries were optimized using the conjugate gradient method until the residual forces on the atoms were smaller than 0.01 eV/Å. The criterion for electronic convergence was an energy change below 10^–6^ eV. The surface energies of stoichiometric and symmetric slabs of area *A* were calculated using the equation γ = (*E*_slab_ – *E*_bulk_ × *N*_slab_/*N*_bulk_)/2*A*, where *E*_slab_ and *E*_bulk_ are the total energy of the slab model and the optimized bulk unit cell, respectively, while *N*_slab_ and *N*_bulk_ represent the number of atoms in the slab model and the bulk unit cell, respectively. Adsorption energies per molecule were calculated as the total energy difference between the relaxed combined system and the bare surface and isolated molecules in the gas phase. The energy barrier to form a carbonate was calculated using the climbing image nudged elastic band (CI-NEB) method.^82^ Vibrational frequency analysis was conducted to confirm that the optimized transition state corresponds to a first-order saddle point on the potential energy surface, as evidenced by the presence of a single imaginary vibrational mode. Bader charge analysis was performed using the Bader partitioning method implemented by Henkelman et al.^83^ to quantify atomic charges. Differential charge density plots were obtained by calculating the difference between the total charge density of the combined system (comprising the adsorbed CO_2_ molecule and the substrate) and the sum of the charge densities of the isolated constituents (gas-phase CO_2_ molecule with the same bond lengths and angles as when adsorbed and the substrate with the atomic coordinates taken from the system with the CO_2_ adsorbed). This approach spatially resolves regions of electron density accumulation and depletion, delineating charge redistribution arising from molecule–substrate interactions.

Noncontact AFM images of the relaxed models were simulated with the Probe-Particle Model,^60^ which includes the electrostatic potential above the surface (from the DFT calculation), Lennard-Jones potentials, and the elastic properties of the tip. Cu, CuOx and CO tips were simulated using the following values of lateral and vertical spring constants and charges (Cu: *k*_*x*,*y*_ = 0.75 N/m, *k*_*z*_ = 50.7 N/m, effective tip charge −0.05 *e*; CuOx: *k*_*x*,*y*_ = 161.9 N/m, *k*_*z*_ = 271.1 N/m, effective tip charge −0.05 *e*; CO: *k*_*x*,*y*_ = 1.7 N/m, *k*_*z*_ = 326.9 N/m, effective tip charge −0.005 *e*). The experimental oscillation amplitude was used in the simulations. Since the exact height of the tip in the experiment is unknown, the height yielding the best visual agreement between the experiment and the simulation was chosen.

## Supplementary Material





## Notes

Section S1: (001) vs (100) terminations; Section S2: additional sample characterization (polarization microscope images, XRD data, EBSD data, additional ambient AFM data, additional photographs of the sample, XPS data); Section S3: mass spectrometry during cleaving; Section S4: additional nc-AFM data and AFM simulations (tip functionalization, pristine surface, CO_2_-adsorbed surface); Section S5: additional computational results (DFT-optimized models and AFM simulations, complete Bader charge analysis, atom-projected DOS for physisorbed and chemisorbed CO_2_) (PDF)
Structure file St1: DFT-optimized structure of the pristine wollastonite (100) surface (Figure S12A); Structure file St2: DFT-optimized structure of the hydrated wollastonite (100) surface with nested H_2_O adsorbed (Figures 2A and S12B); Structure file St3: DFT-optimized structure of the hydrated wollastonite (100) surface with nested H_2_O and CO_2_ adsorbed (Figures 3A and S12E); Structure file St4: DFT-optimized structure of the pristine wollastonite (100) surface with nested CO_2_ adsorbed (Figure S12D); Structure file St5: DFT-optimized structure of the pristine wollastonite (100) surface with nested CO_2_ and additional CO_2_ adsorbed (Figure S12F) (ZIP)

## Abbreviations

CI-NEB: climbing image nudged elastic band
DFT: density functional theory
DOS: density of states
EBSD: Electron backscattered diffraction
FFT: fast Fourier transform
Nc-AFM: noncontact atomic force microscopy
(sc)­CO_2_: supercritical carbon dioxide
STM: scanning tunneling microscopy
UHV: ultrahigh vacuum
XPS: X-ray photoelectron spectroscopy
XRD: X-ray diffraction
