# Supplementary material for: Molecular Views of Mineral Carbonation: Reaction of CO2 with the Wollastonite (100) Surface
Source: ACS Nano. 2026 Mar 24;20(13):10456–65. doi: 10.1021/acsnano.5c19629 (PMC13063804; doi:10.1021/acsnano.5c19629)
Supplement: Supplementary file 1 [file nn5c19629_si_001.pdf]

Supplementary Information:
Molecular views of mineral carbonation: reaction of CO_2 with the wollastonite (100) surface

Andrea Conti,[1‡] Luca Lezuo,[1‡] Alexander Hoheneder,[1] Elena Vaníčková,[2] Domitilla Alessandra Aloi,[1] Andreas Steiger-Thirsfeld,[3] David Heuser,[4] Rainer Abart,[4] Florian Mittendorfer,[1] Michael Schmid,[1] Ulrike Diebold,[1] Giada Franceschi[1]*

[1]Institute of Applied Physics, TU Wien, 1040, Vienna, Austria

[2]Central European Institute of Technology, Brno University of Technology, 61200, Brno, Czech Republic

[3]University Service Center for Transmission Electron Microscopy, TU Wien, 1040, Vienna, Austria

[4]Department of Lithospheric Research, Universität Wien, 1090, Vienna, Austria

This PDF file includes:

Section S1: (001) vs. (100) terminations 2

Section S2: Additional sample characterization 3

Polarization microscope images 3

XRD data 4

SEM and EBSD data 5

Additional ambient AFM data 6

Additional photographs of the sample 7

XPS data 7

Section S3: Mass spectrometry during cleaving 8

Section S4: Additional nc-AFM data and AFM simulations 9

Tip functionalization 9

Pristine surface 10

CO_2-adsorbed surface 11

Section S5: Additional computational results 12

DFT-optimized models and AFM simulations 12

Complete Bader charge analysis 13

Atom-projected DOS for physisorbed and chemisorbed CO_2 14

Section S1: (001) vs. (100) terminations

In the literature, there is some disagreement regarding whether the most stable surface of wollastonite 1A should be labelled as (100) or (001). Below, it is argued that this disagreement might originate from a difference in lattice convention.

Wollastonite-1A crystallizes in a triclinic unit cell that can be described using two alternative notations. The conventional unit cell (commonly used in mineralogical literature and in the scientific literature[1–3]) adopts $a > b > c$, whereas computational databases (Materials Project, NOMAD) and other recent literature[4] use a reduced cell with $a < b < c$. In practice, this corresponds to an exchange of the a and c lattice vectors and, simultaneously, of the α and γ angles. As a consequence, the (100) and (001) planes are interchanged between the two notations. The conventional unit cell, resulting in the labelling of the lowest-energy surface as (100), was adopted throughout this work (both main text and SI).

The wollastonite surfaces cleaved in this work consist of the lowest-energy (100) facet (conventional-unit-cell notation), as determined by EBSD analysis (Figure S3). This behavior is expected from the cleavage behavior of wollastonite-1A: (001) is reported as a "good" cleavage plane, whereas (100) is described as "perfect".[5] Consistently, density functional theory calculations yield surface energies of 82 meV/Å² and 58 meV/Å² for the (001) and (100) terminations, respectively. The geometric analysis from all acquired nc-AFM images is also consistent. As shown in Figure 1A, the surface unit cell of the (001) termination is an oblique parallelogram, with an internal angle of 103°. In contrast, the (100) termination has an almost rectangular surface unit cell (α = 90.055°, see Methods Section of the main text). The experimental nc-AFM images acquired at LHe temperatures show angles of 90°±3° (Figures 1E and 2B; deviations of more than 10° are excluded), consistent with the (100) termination.

Section S2: Additional sample characterization

Polarization microscope images

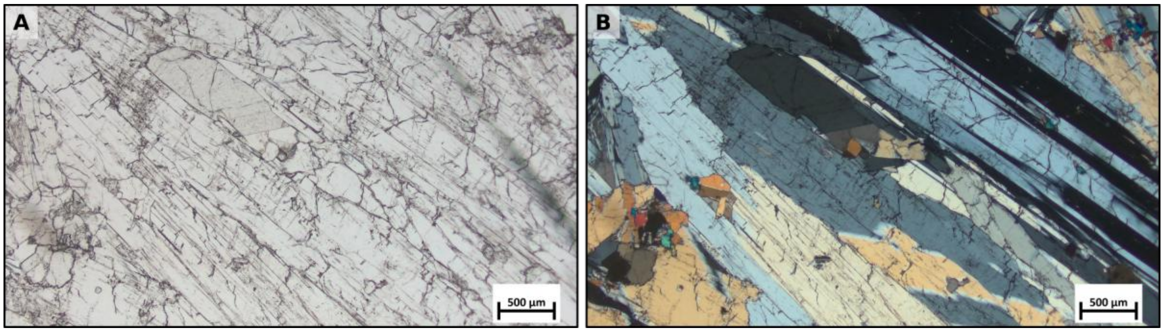

Figure S1. Thin section (thickness 30 µm) of an aggregate of several ca. 100 µm wide and several millimeter long rod- or lath-shaped wollastonite grains under (A) plane-polarized light and (B) cross-polarized light. In cross-polarized light (B), the grains display low first-order interference colors (black, grey, pale yellow) according to the Michel-Lévy chart, reflecting the low birefringence[6] characteristic of wollastonite. The color variations arise from different crystallographic orientations or twin domains within individual grains. The elongation direction (top left to bottom right) corresponds to the [010] direction. The pale blue color shades result from the application of a blue color filter. The thin straight lines parallel to the elongation direction are traces of the (100) and (102) cleavage planes.

XRD data

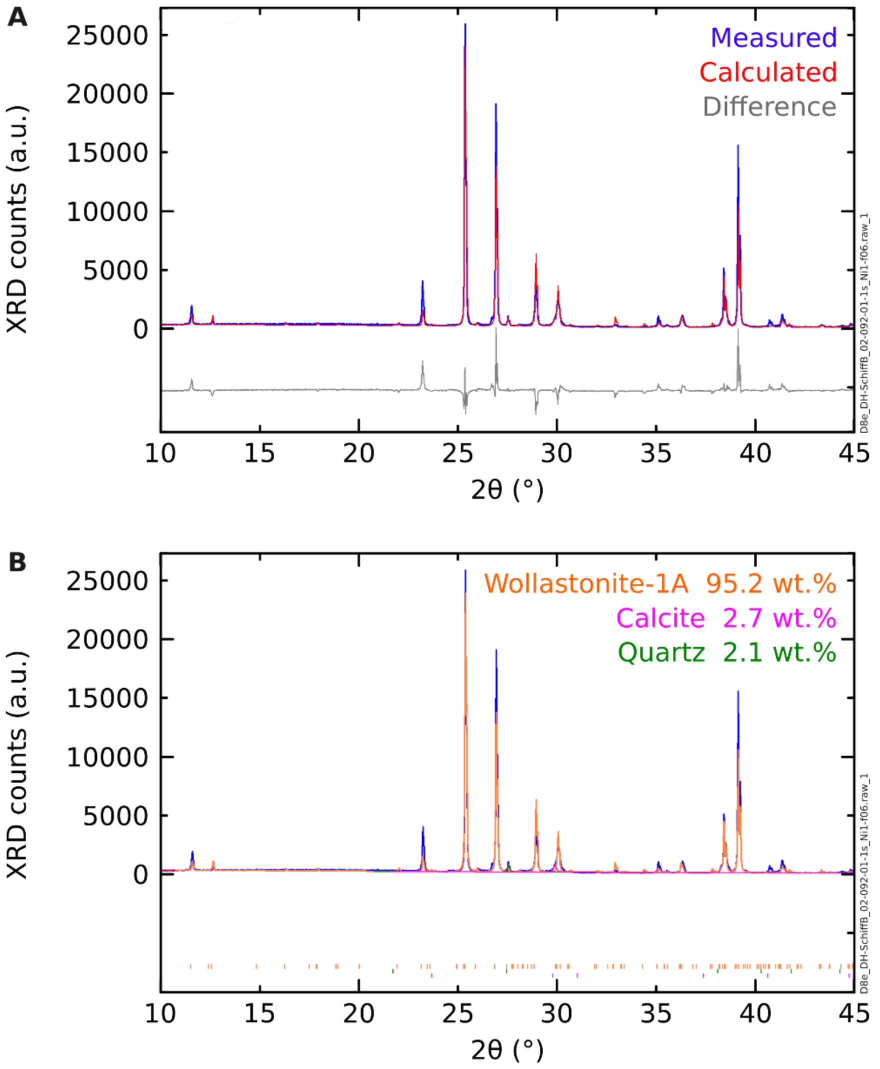

Figure S2. X-ray diffraction (XRD) patterns and Rietveld refinement results for the sample material. (A) The plot displays the measured pattern (blue), the calculated model (red), and the difference curve (grey). (B) Quantitative phase analysis indicates a composition of wollastonite-1A (95.2 wt.%), calcite (2.7 wt.%), and quartz (2.1 wt.%). Vertical tick marks at the bottom indicate the Bragg reflection positions for wollastonite (orange), calcite (magenta), and quartz (green). The mismatch in peak intensities (visible in the difference curve of panel A) is attributed to preferred orientation effects caused by the alignment of acicular wollastonite crystallites. Despite these texture effects, the primary phase is unambiguously identified as triclinic wollastonite-1A with lattice parameters a = 7.9258 Å, b = 7.3202 Å, c = 7.0653 Å, α = 90.055°, β = 95.217°, γ = 103.426°.

SEM and EBSD data

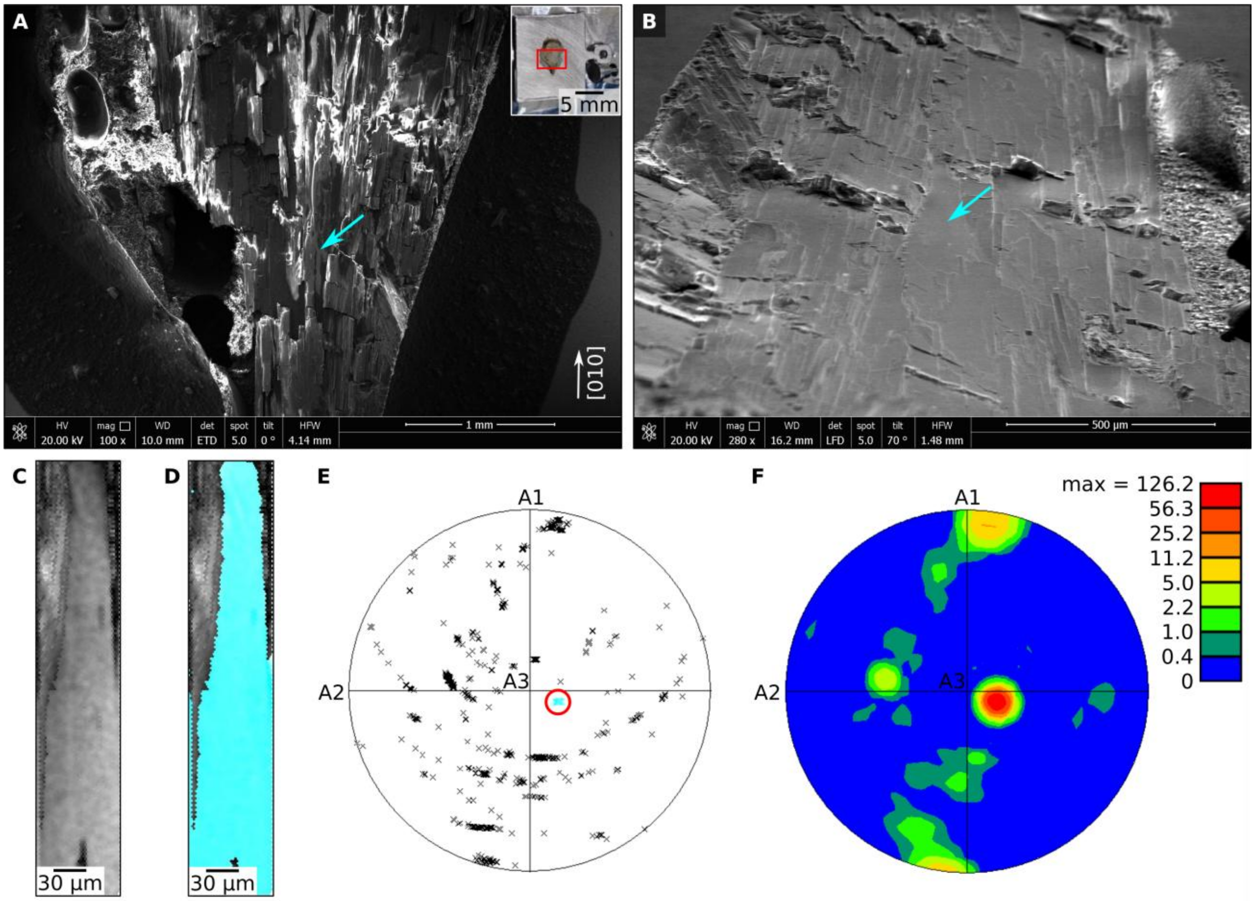

Figure S3. (A, B) Scanning electron microscopy (SEM) secondary electron images of the same wollastonite sample photographed in Figure S5. The red rectangle in the inset of panel A indicates the field of view for the main SEM image. Using the image of the UHV-cleaved sample as a reference, it was possible to pinpoint the location of the nc-AFM tip in UHV and perform electron backscattered diffraction (EBSD) measurements in this region (cyan arrows). The sample was rotated by 180° between panel A and the subsequent panels to minimize topographic shadowing during EBSD acquisition. (C–F) EBSD analysis: (C) Image of the selected terrace. (D) Corresponding EBSD orientation map demonstrating the uniform crystallographic orientation of the measured region (indicated by the single cyan color). (E) Discrete {100} pole figure obtained from the area in panel (C). A1, A2, and A3 define the sample reference coordinate system; A1 and A2 lie in the plane of the sample holder, while A3 is normal to it. The {100} pole of the face of interest (circled in red) points nearly perpendicular to the sample holder, confirming that the exposed surface of the measured terrace is the (100) plane. The slight deviation from the center is likely due to the measured surface not being perfectly parallel to the sample holder. Such a tilt was also observed during the nc-AFM measurements and is likely due to the uneven morphology of the back of the sample, which affected its mounting to the sample plate. (F) Contoured {100} pole figure calculated from the discrete orientation data of panel E. The pole density was modeled using a harmonic series expansion of rank 34 with a 6.0° Gaussian smoothing and triclinic sample symmetry. The high-intensity region (red) corresponds to the primary cluster of orientations in the discrete plot.

Additional ambient AFM data

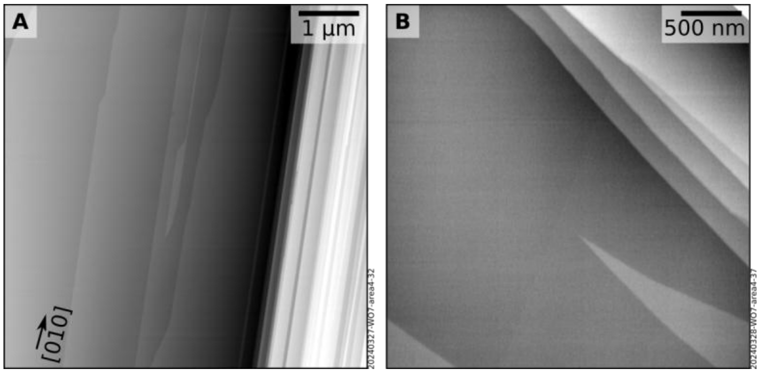

Figure S4. Additional ambient AFM images of cleaved wollastonite. (A) 6 × 6 µm^2; (B) 3 × 3 µm^2.

Additional photographs of the sample

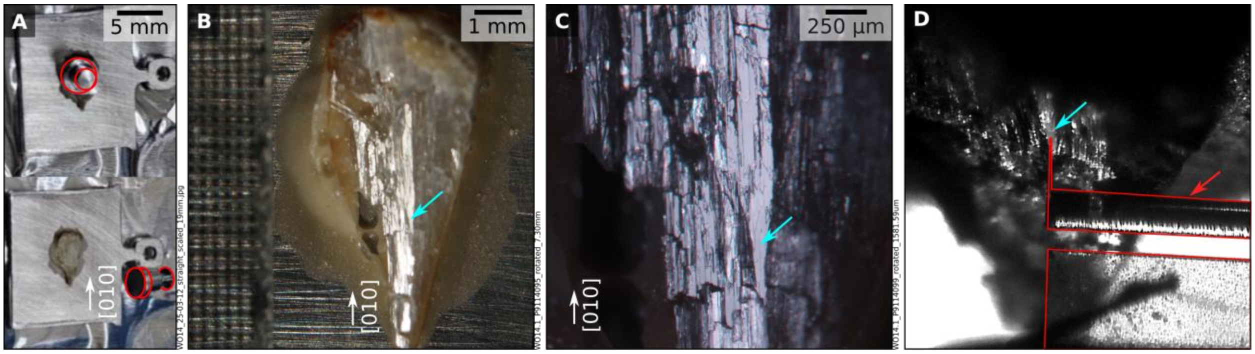

Figure S5. (A) Wollastonite glued on a sample plate with a stud (outlined in red), before (top) and after (bottom) cleaving. (B and C) Additional optical characterization. Macroscopically flat terraces reflect incident light, appearing as bright regions. (D) Photograph of the qPlus sensor (red arrow and outline) approached to a macroscopically flat surface in UHV. Cyan arrows indicate the specific terrace measured by EBSD (Figure S3) and nc-AFM (panel D).

XPS data

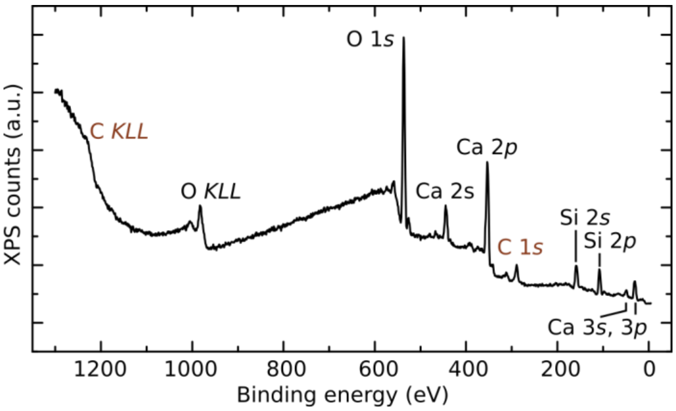

Figure S6. XPS survey of UHV-cleaved wollastonite. Normal emission (Al Kα, 1486.61 eV, pass energy 60 eV). The binding energy axis was adjusted to account for charging by setting the C 1s binding energy to 285 eV. The strong C signal may be due to the glue used for mounting, calcite inclusions, and/or surface contamination in portions of the wollastonite needles.

Section S3: Mass spectrometry during cleaving

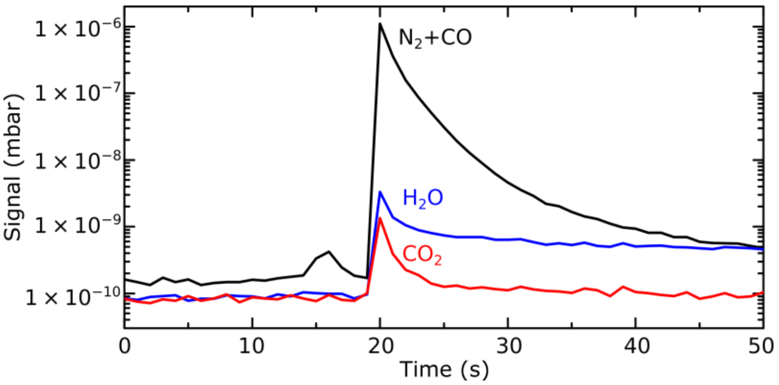

Figure S7. Partial pressures of masses 28 (N_2 + CO), 44 (CO_2), and 18 (H_2O) (logarithmic scale) measured by a mass spectrometer while cleaving a wollastonite grain in a UHV chamber at room temperature. The chamber is continuously pumped, leading to a pressure spike caused by cleaving. The effective water vapor dose at the sample surface is likely higher than that measured by the mass spectrometer, which is positioned in a different region of the chamber than the cleaving site. Note that only H_2O is expected to adsorb at room temperature under low-pressure conditions.

Section S4: Additional nc-AFM data and AFM simulations

Tip functionalization

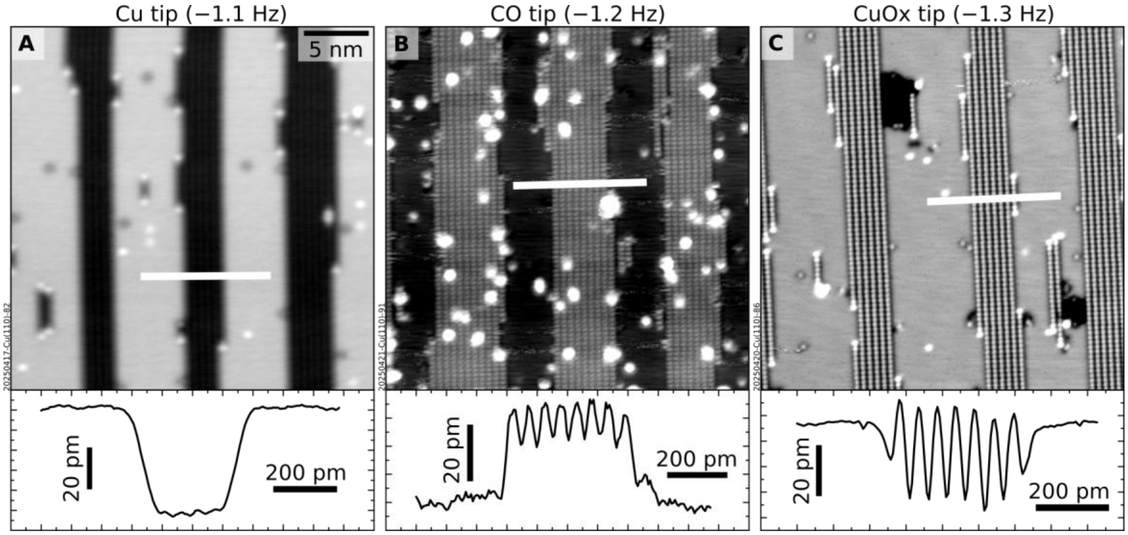

Figure S8. Tip identification on (2 × 1)-O-reconstructed Cu(110), after a procedure introduced by the Mönig group.[7] From left to right: STM images acquired with Cu-, CO-, and CuOx-terminated tips. The frequency shifts at the imaging setpoint (V_s = 500 mV, I_t = 100 pA) are given at the top. Characteristic line profiles help identifying the tip termination.

Pristine surface

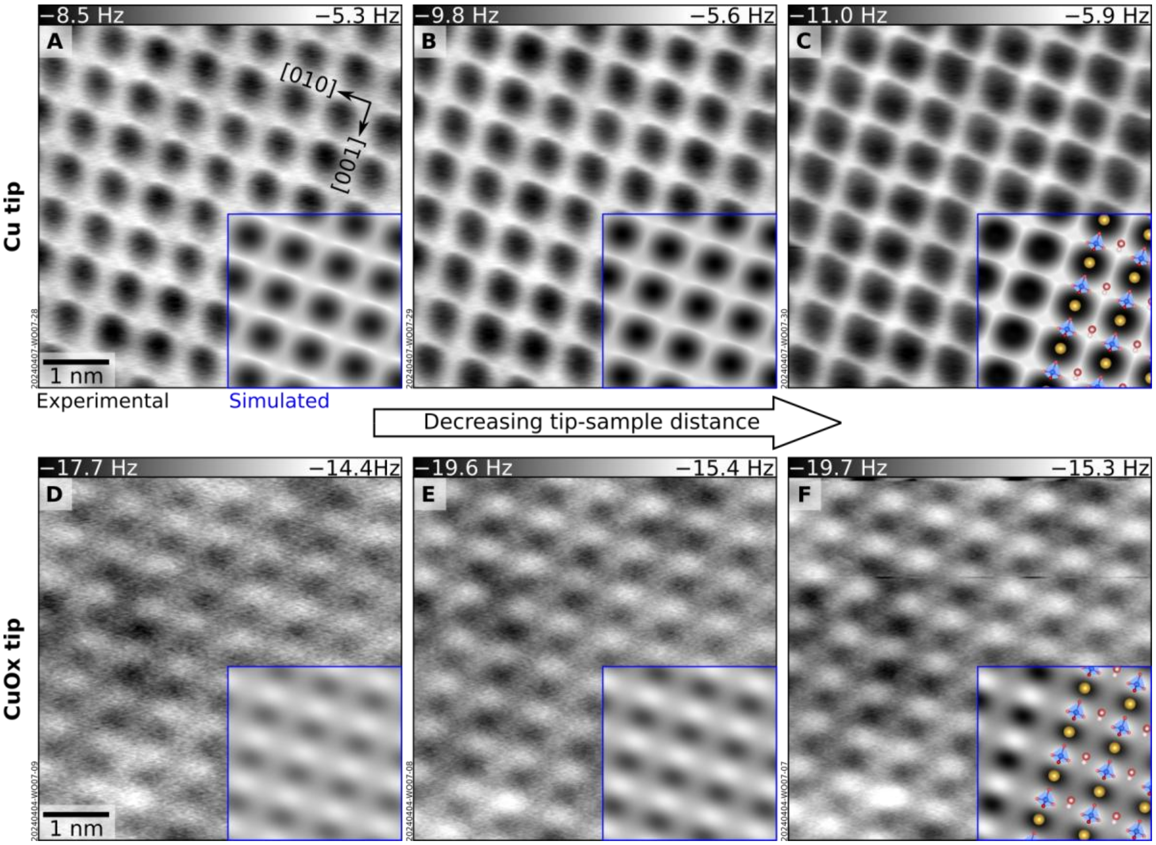

Figure S9. 5.5 × 5.5 nm^2 nc-AFM images of the cleaved wollastonite surface as a function of tip-sample distance with Cu- and O-terminated tips acquired at 5.7 K (A = 200 pm, V_s = – 10 V). Insets (with blue frames): DFT-based simulations with the Probe-Particle Model[8] for tip-sample distances of (A and D) 6.4 Å, (B and E) 6.0 Å, and (C and F) 4.8 Å.

CO_2-adsorbed surface

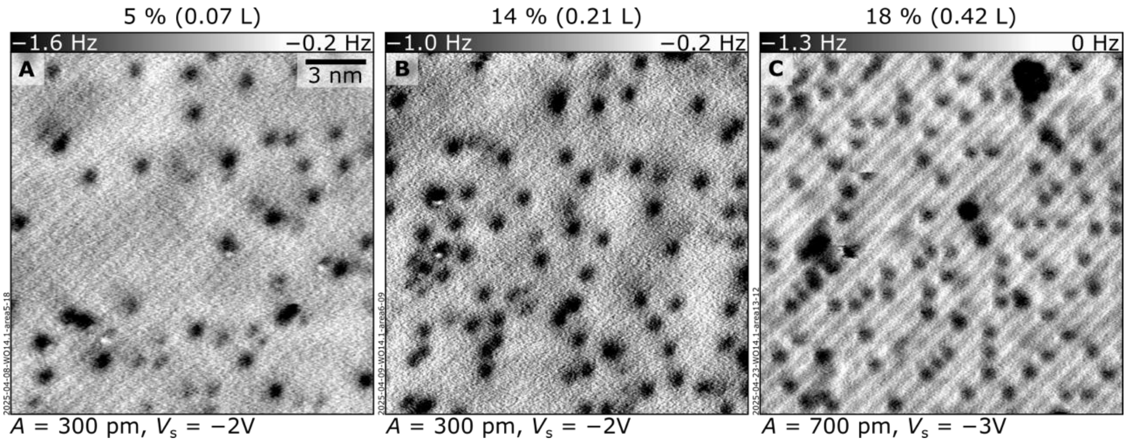

Figure S10. Overview of increasing CO_2 coverage. 18 × 18 nm^2 nc-AFM images acquired with Cu-terminated tips at 78 K. The percentages of lattice sites occupied by CO_2 molecules and corresponding doses in Langmuir are indicated at the top.

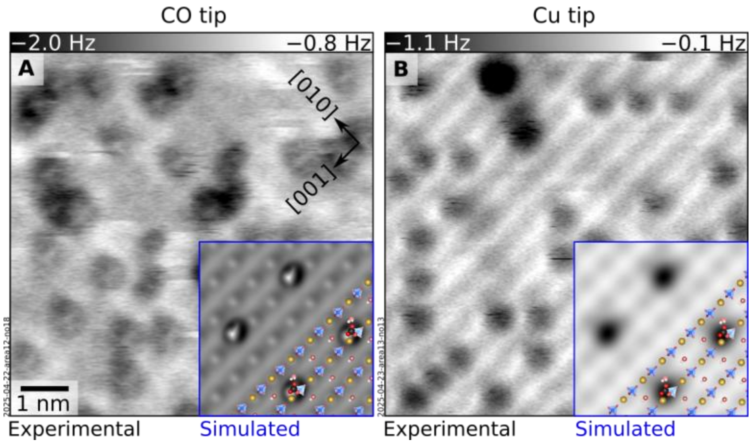

Figure S11. Nc-AFM images of CO_2 adsorbed on UHV-cleaved wollastonite (100) with CO- and Cu-terminated tips and corresponding DFT-based simulations (overlays with blue outlines). The experimental images were acquired at 78 K (A = 700 pm, V_s = – 3 V).

Section S5: Additional computational results

DFT-optimized models and AFM simulations

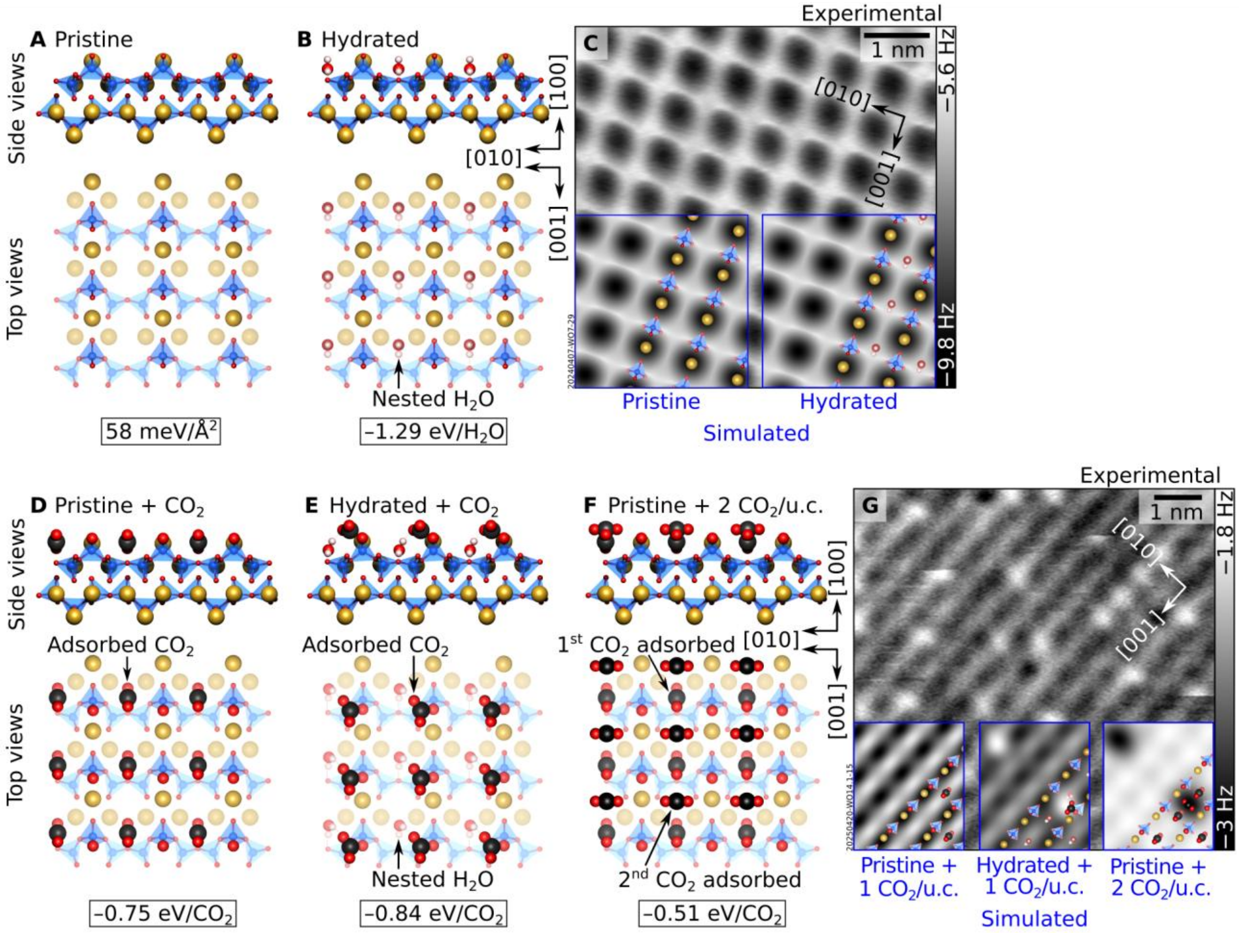

Figure S12. (A and B) DFT-optimized models of the pristine (water-free) and hydrated (1 H_2O/u.c.) surface, respectively. (C) Nc-AFM image of UHV-cleaved wollastonite (100), adapted from the main text. Insets: simulated AFM images of the DFT-optimized structures in panels A and B. (D–F) DFT-optimized models of (D) the pristine surface after the adsorption of 1 CO_2/u.c., (E) the hydrated surface after the adsorption of 1 CO_2/u.c., and (F) the pristine surface after the adsorption of 2 CO_2/u.c. (G) Nc-AFM image of CO_2 adsorbed on UHV-cleaved wollastonite (100), adapted from the main text. Insets: simulated AFM images of the DFT-optimized structures in panels D–F (the simulations are obtained for CO_2 coverages comparable to the experiments, ≈ 15% of occupied lattice sites). Without nested water, neither the 1st nor the 2nd CO_2 molecule per unit cell can explain the experimental nc-AFM images. Ca, Si, O, H and C atoms are shown in dark yellow, blue, red, white and black, respectively.

Complete Bader charge analysis

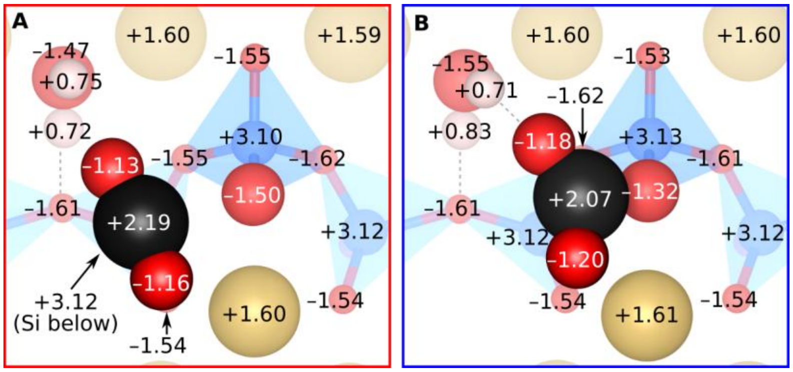

Figure S13. Bader charges (A) in the physisorbed state and (B) for chemisorbed CO_2 on hydrated (100) wollastonite. Electron density shifts from the hydrated surface to the adsorbed CO_2, converting it to a more negatively charged carbonate-like species. Ca, Si, O, H, and C atoms are shown in dark yellow, blue, red, white, and black, respectively.

Atom-projected DOS for physisorbed and chemisorbed CO_2

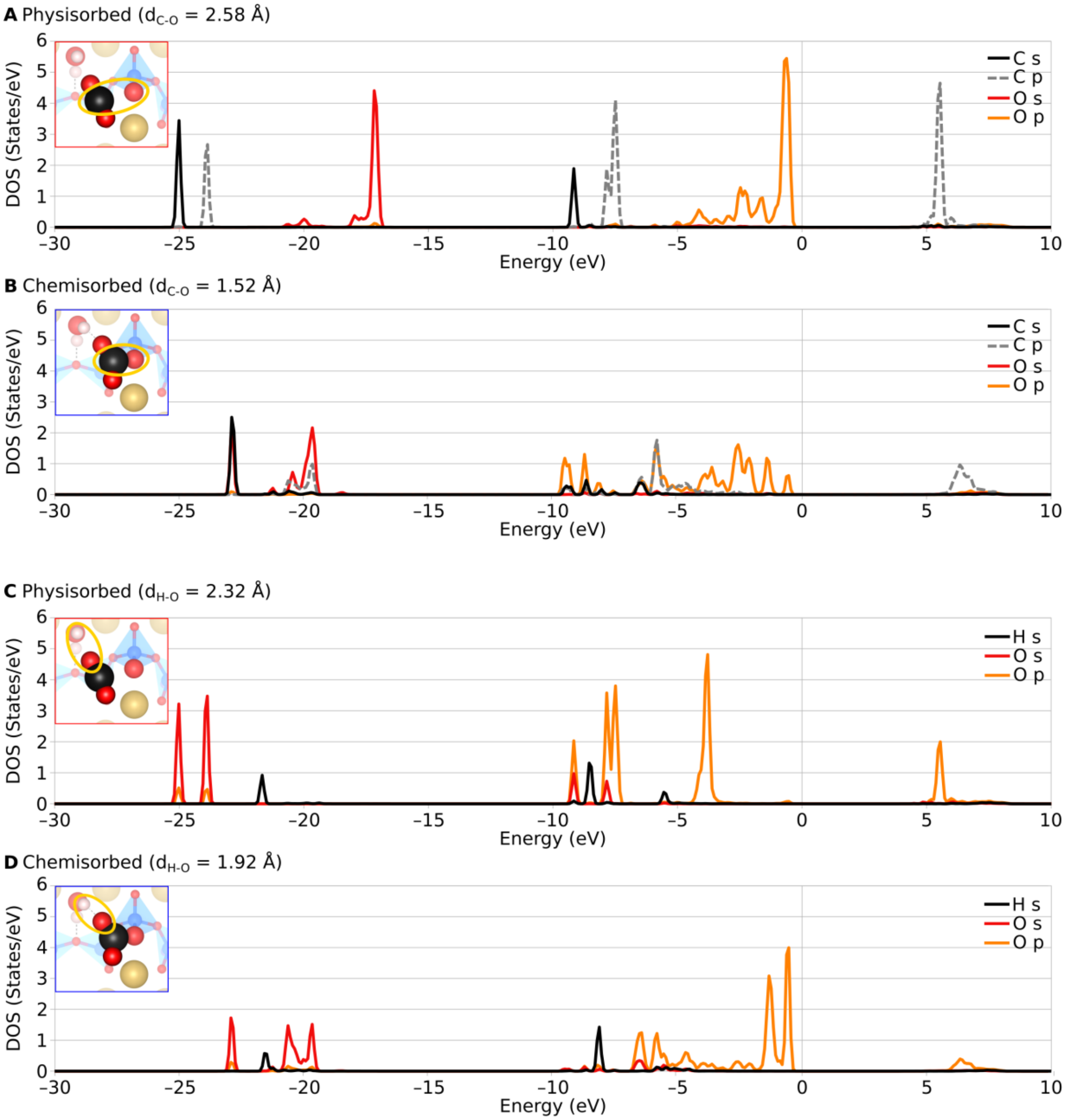

Figure S14. Atom-projected DOS for physisorbed and chemisorbed CO_2. Insets at the left highlight the atoms whose DOS is shown. (A) In the physisorbed state, the C 2s and C 2p peaks are well separated from the O 2s and O 2p levels of the substrate, with negligible C p–O p overlap. (B) Upon chemisorption, the deep σ-states shift and hybridize slightly, and new states appear between −10 and 0 eV carrying similar C 2p and O 2p weight. The shared C p–O p confirms 2p–2p hybridization and covalent C–O bond formation in the surface carbonate. (C) In the physisorbed state, the H 1s peaks do not substantially hybridize with the O 2s and O 2p levels. (D) Upon chemisorption, the deep σ-states shift; the overlap of the H and O states between −21 and −19 eV and −8 and 0 eV is consistent with the formation of an H-bond between the protruding H of the nested H_2O and the closest O of the chemisorbed CO_2. Distances between atoms are shown in brackets.

References

(1) Kundu, T. K.; Hanumantha Rao, K.; Parker, S. C. Atomistic Simulation of the Surface Structure of Wollastonite and Adsorption Phenomena Relevant to Flotation. *Int. J. Miner. Process.* **2003**, *72* (1–4), 111–127. https://doi.org/10.1016/S0301-7516(03)00092-9.

(2) Ito, T.; Sadanaga, R.; Takeuchi, Y.; Tokonami, M. The Existence of Partial Mirrors in Wollastonite. *Proc. Jpn. Acad.* **1969**, *45* (10), 913–918. https://doi.org/10.2183/pjab1945.45.913.

(3) Buerger, M. J.; Prewitt, C. T. The Crystal Structures of Wollastonite and Pectolite. *Proc. Natl. Acad. Sci.* **1961**, *47* (12), 1884–1888. https://doi.org/10.1073/pnas.47.12.1884.

(4) Thissen, P.; Natzeck, C.; Giraudo, N.; Weidler, P.; Wöll, C. Hydration of Concrete: The First Steps. *Chem. Eur. J.* **2018**, *24* (34), 8603–8608. https://doi.org/10.1002/chem.201705974.

(5) *Handbook of Mineralogy*; Anthony, J. W., Bideaux, K. W., Bladh, K. W., Nichols, M. C., Eds.; Mineralogical Society of America: Chantilly, VA 20151-1110, USA.

(6) Wahlstrom, P. G. E. E. *Optical Crystallography*, 5th ed.; Wiley: New York, 1979. https://doi.org/10.1017/S0016756800033379.

(7) Schulze Lammers, B.; Yesilpinar, D.; Timmer, A.; Hu, Z.; Ji, W.; Amirjalayer, S.; Fuchs, H.; Mönig, H. Benchmarking Atomically Defined AFM Tips for Chemical-Selective Imaging. *Nanoscale* **2021**, *13* (32), 13617–13623. https://doi.org/10.1039/d1nr04080d.

(8) Oinonen, N.; Yakutovich, A. V.; Gallardo, A.; Ondráček, M.; Hapala, P.; Krejčí, O. Advancing Scanning Probe Microscopy Simulations: A Decade of Development in Probe-Particle Models. *Comput. Phys. Commun.* **2024**, *305*, 109341. https://doi.org/10.1016/j.cpc.2024.109341.
